# Rescuing and utilizing anticancer *Nothapodytes* species: Integrated studies from plant resources to natural medicines

**DOI:** 10.1002/ctm2.70110

**Published:** 2024-11-26

**Authors:** Xingrong Peng, Xianghai Cai, Jia Tang, Jia Ge, Gao Chen

**Affiliations:** ^1^ Key Laboratory of Phytochemistry and Natural Medicines, Kunming Institute of Botany Chinese Academy of Sciences Kunming China; ^2^ State Key Laboratory of Phytochemistry and Plant Resources in West China, Kunming Institute of Botany Chinese Academy of Science Kunming China; ^3^ Key Laboratory for Plant Diversity and Biogeography of East Asia, Kunming Institute of Botany Chinese Academy of Sciences Kunming China; ^4^ Yunnan Key Laboratory for Integrative Conservation of Plant Species with Extremely Small Populations Kunming China

Cancer remains the second leading cause of death globally, following cardiovascular disease, and represents a major public health challenge.^1^ It arises from extensive DNA damage induced by ultraviolet radiation, ionizing radiation, environmental factors, and therapeutic agents. Among cancer types, the most frequently diagnosed are lung (12.7%), breast (10.9%), colorectal (9.7%) and gastric cancers (7.8%).^2^ In China, lung cancer accounts for the highest cancer mortality, with 657 000 deaths (27.2%) and a crude mortality rate of 47.5 per 100 000 individuals.^3^ According to the World Health Organisation, cancer contributes to the largest global disease burden, with 244.6 million disability‐adjusted life years (DALYs) lost, affecting both men (137.4 million DALYs) and women (107.1 million DALYs).^4^ Consequently, there is an urgent need for more effective medications and therapeutic strategies to reduce mortality, minimize side effects, and improve patient prognosis.

While conventional anticancer treatments, such as surgery, radiotherapy and hormonal therapy, have advanced, the field of cancer therapeutics is intensively focused on improving survival outcomes.^5^ Emerging therapies, including immunotherapy, gene therapy, and molecular‐targeted treatments, show particular promise in enhancing efficacy.^5^ Among these, molecular‐targeted therapy has gained attention for its ability to directly interfere with oncogenic molecules, effectively blocking sites critical to cancer progression.^6^ Over the past decade, antibody‐drug conjugates (ADCs), which combine a monoclonal antibody with a cytotoxic drug linked by a chemical bridge, have achieved substantial success, due to their highly selective delivery of toxic agents to cancer cells.^7^ Consequently, exploring diverse therapeutic strategies remains essential for advancing anticancer drug development.

Small‐molecule drugs play a critical role in chemotherapy for treating malignant and metastatic diseases. Over 60% of current anticancer drugs originate from natural sources, including plants, animals and microbes.^8^ For instance, vinblastine and vincristine from *Catharanthus roseus* (Apocynaceae), taxol and docetaxel from *Taxus* species (Taxaceae) and camptothecin (CPT) from *Camptotheca acuminata* (Nyssaceae) are among the most effective cancer chemotherapeutics available today.^9^ However, the primary challenge in developing nature‐derived anticancer drugs is the limited availability of natural resources. Taxol, known for its high efficacy, low toxicity and broad‐spectrum activity, is a textbook case of this challenge. To meet demand, large quantities of *Taxus* trees have been felled. This substantially contributed to the depletion of these resources, which is critical since these trees have a long developmental cycle. Currently, all 11 *Taxus* species are listed on the International Union for Conservation of Nature Red List of Endangered Species as of 2013.

Camptothecin is a monoterpene indole alkaloid that specifically targets DNA topoisomerase I and is recognized as the third most significant anticancer drug after taxol and vinblastine.^10^ With a total trade volume exceeding $10 billion, CPT continues to be a focus for the development of potent and safer anticancer drugs. Modifications to CPT's structure have produced several derivatives, such as irinotecan, topotecan, and belotecan, with enhanced antitumor activity and improved stability, achieved by introducing hydrophilic amino and hydroxyl groups to the A/B rings of CPT.^11^ Additionally, CPT is increasingly utilized as a payload in ADCs and small‐molecule drug conjugates, optimizing its therapeutic potential.^12^

Because *Nothapodytes* species contain 3–7 times more CPT than *Camptotheca acuminata*, the genus *Nothapodytes* has become the primary source of CPT since 2003. However, the nine *Nothapodytes* species, primarily distributed across tropical regions of southern and southeastern Asia, have been overexploited. In particular, over 80% of *Nothapodytes* plants in Southwest China have been depleted, with only a small number of plants now surviving in protected natural areas (unpublished data). Thus, we urgently call for the conservation, protection and sustainable use of all *Nothapodytes* species to preserve these valuable anticancer resources.^1^

To conserve and effectively utilize anticancer *Nothapodytes* species, we present a comprehensive framework for designing integrated studies from plant resources to natural medicines (Figure 1). This framework can also be applied to other plant resources facing similar challenges with high demand for natural medicine discovery yet enviromental susceptibility. We focus on six avenues essential to ensure a durable and sustainable supply of CPT from *Nothapodytes* trees.

1. **Reproductive biology**: Factors like flowering time, pollinator interactions and seed dispersal mechanisms influence the regeneration and recovery potential of plant populations under exploitation. Understanding how bioactive compound production impacts plant reproductive success is essential for developing effective conservation strategies.^13^
2. **Spatio‐temporal distribution**: Studying the spatial and temporal distribution of *Nothapodytes* plant populations, along with taxonomic clarification and genetic diversity assessments, is vital to ensure conservation efforts focus on the most vulnerable populations. These studies lay the foundation for region‐specific conservation strategies that consider both the ecological and pharmacological importance of these plants.
3. **Omics approaches**: Genomic, population genomic, transcriptomic and metabolomic approaches reveal genetic and metabolic diversity, enabling the identification of genes and pathways involved in synthesizing bioactive compounds.^14^, ^15^
4. **Defence and detoxification dynamics**: Plants produce secondary metabolites to defend themselves against biotic and abiotic stressors, while herbivores and pathogens evolve detoxification mechanisms that they can use gut enzymes or gut microbes to modify secondary metabolites (toxins). Leveraging this “defence and detoxification” dynamics can yield structurally diverse molecules.^16^
5. **Symbiosis**: Symbiosis, particularly with bacteria and fungi, is another avenue for discovery. In some cases, lateral gene transfer has led symbiotic organisms to acquire plant genes, enabling them to produce similar secondary metabolites. For example, fungi such as *Aspergillus*, *Trichoderma*, *Fomitopsis*, *Phomopsis* and *Fusarium* have been found to produce CPT.^15^
6. **Synthetic modifications**: Isolated natural products serve as models for developing more effective analogues and prodrugs. Synthetic methodologies, including total and combinatorial synthesis, are crucial for creating structurally diverse molecules with potent pharmacological effects. Advanced synthetic strategies such as Function‐Oriented Synthesis,^17^ Biology‐Oriented Synthesis,^18^ Diversity‐Oriented Synthesis^19^ and Pharmacophore‐Oriented SemiSynthesis^20^ should be prioritized in future research.

**FIGURE 1:**
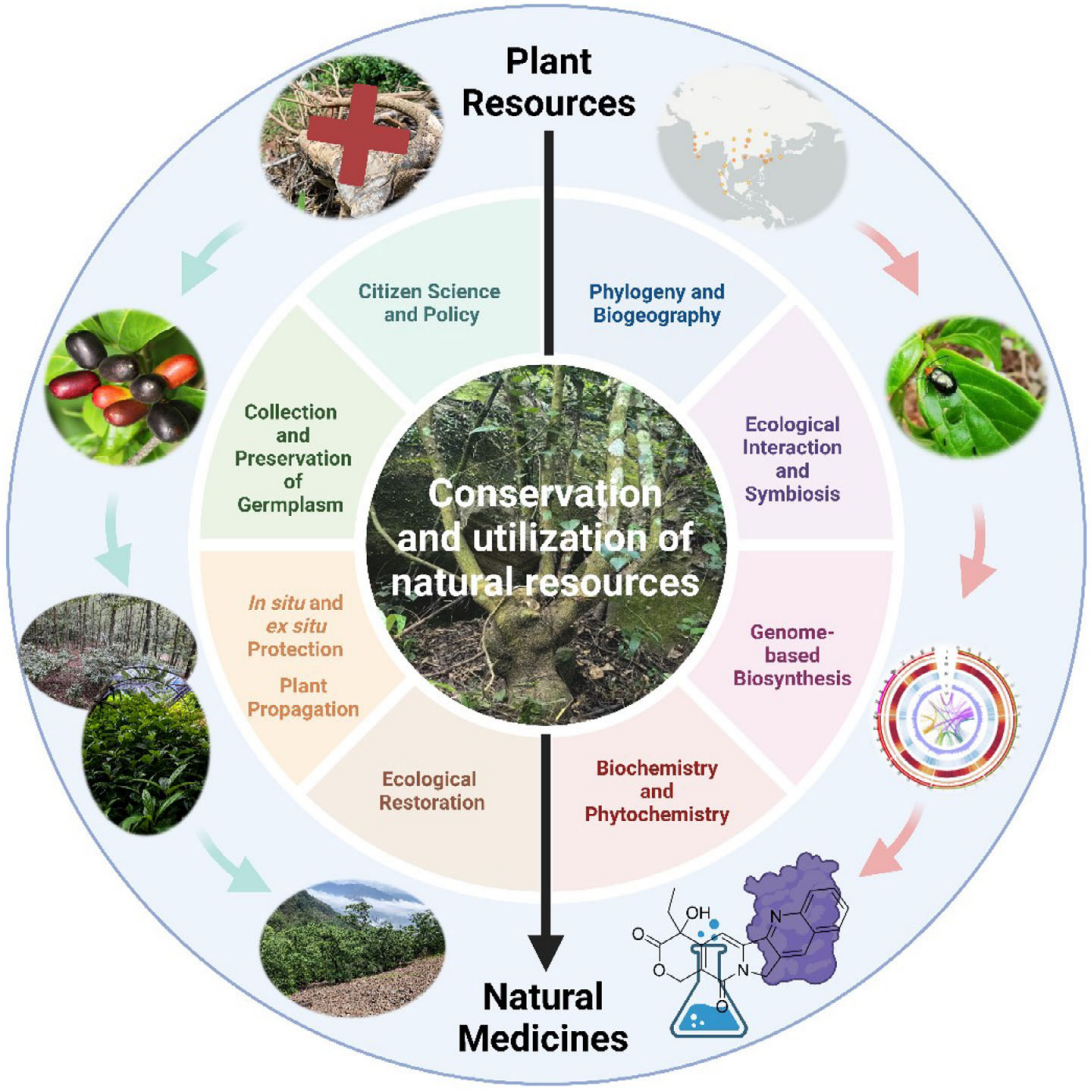
Framework for the conservation and sustainable utilization of plant resources (a chain from plant resources to natural medicines).

In conclusion, conserving threatened medicinal plant species with broad distribution requires a multifaceted approach. We should prioritize studies on plant resources within target taxa, encompassing biogeography, systematics, and distribution. Equally important is understanding the diversity and accumulation mechanisms of active compounds across spatial and temporal scales, alongside photosynthetic and physiological influences on target compound production. Additionally, assessing the ecological role of active compounds under biotic or abiotic pressures, including symbiotic interactions with endophytic bacteria or fungi that may contribute to compound synthesis, is essential. Investigating the biosynthesis pathways of these compounds, as well as their pharmacokinetics and pharmacodynamics, will further support conservation efforts.

Moreover, policy standards must be established to protect these target species, alongside evaluating their conservation status and enhancing sustainable plant resource supply. Strengthening the pharmaceutical development pipeline, from plant resources to active ingredients, lead compounds, and natural medicines, will be crucial for integrating conservation with advances in pharmacology (Figure 1).

## AUTHOR CONTRIBUTIONS

*Conceptulization*: Gao Chen, Xianghai Cai, Jia Tang, Jia Ge. *Formal analysis and investigation*: Gao Chen, Xingrong Peng. *Writing—original draft preparation*: Xingrong Peng. *Supervision*: Gao Chen.

## CONFLICT OF INTEREST STATEMENT

The authors declare no conflict of interest.
